# Postpartum depression and its associated risk factors among tribal females attending rural health training centre

**DOI:** 10.6026/973206300212425

**Published:** 2025-08-31

**Authors:** Umashree Sharma, Dhruvendra Pandey, Nitish Adawadkar, Anand Kumar Patidar, Likhar S.K.

**Affiliations:** 1 Department of Community Medicine, Government Medical College, Ratlam, Madhya Pradesh, India

**Keywords:** Postpartum depression, tribal females, maternal health

## Abstract

Postpartum depression (PPD) is a prevalent yet frequently unrecognized mental health condition, especially in marginalized groups such as tribal communities. Consequently, it is important to examine the prevalence and related risk factors of PPD among tribal women visiting a Rural Health Training Centre (RHTC) in Central India. This cross-sectional research involved 100 tribal women who gave birth at RHTC from February to July 2024, utilizing the Edinburgh Postnatal Depression Scale (EPDS) and a structured questionnaire. The study found that 12% of women exhibited PPD (EPDS >13), with the majority of participants aged between 20 and 29 years, 45% lacking formal education and 44% involved in agriculture. Key risk factors identified included gravida, parity, the number of live children and the total number of children, while no significant links were observed with husbands' smoking, alcohol consumption, domestic violence, or the birth of a female child.

## Background:

Postpartum depression (PPD) is a major public health concern affecting mothers during one of the most vulnerable periods of their lives. The World Health Organization identifies maternal mental health as a crucial aspect of reproductive health, especially in low-and middle-income countries (LMICs) where resources are limited and cultural stigmas prevail [1]. Globally, the proportion of PPD is estimated to be 17.2%, with significantly higher rates in Low middle income countries as compared to high-income countries [2]. In India, mental health disorders among women are often underdiagnosed due to stigma, gender norms and lack of awareness [3]. PPD can have detrimental effects not only on the mother but also on infant development and family dynamics [4]. Women suffering from postpartum depression (PPD) are at a higher risk of stopping breastfeeding and may experience difficulties in bonding with their child [5]. They also report lower quality of life [6]. PPD has been linked to increased risk of chronic depression [7]. It is associated with adverse behavioural outcomes in children later in life [8]. National programs such as RMNCH+A and the Mental Healthcare Act (2017) provide frameworks for maternal care; mental health integration is still evolving [9]. Barriers such as poor mental health literacy and limited trained healthcare providers delay diagnosis and treatment in rural and tribal populations [10]. Cultural stigma further hinders recognition and care-seeking [11]. Research shows that socioeconomically disadvantaged and tribal groups are more vulnerable due to restricted access to healthcare, early marriages, high birth rates, anaemia and nutritional deficiencies [12]. Social determinants including domestic violence, poverty and lack of social support increase PPD risk in these groups [13]. Evidence suggests that culturally sensitive community-based interventions and task-shifting to trained community health workers improve early identification and management of PPD in resource-poor settings [14]. However, data on the prevalence and risk factors of PPD specifically among tribal women remain scarce, limiting the development of targeted programs [15]. Therefore, it is of interest to show the proportion and associated risk factors of postpartum depression (PPD) among tribal females attending a Rural Health Training Centre in Central India, to support targeted interventions and inclusive maternal health policies.

## Methodology:

This cross-sectional analytical study was conducted over a six-month period from February to July 2024 in the catchment area of the Rural Health Training Centre (RHTC), which is affiliated with Government Medical College, Ratlam, Madhya Pradesh, India. This region predominantly serves tribal communities, which are often underserved and face unique health challenges. The study aimed to assess the prevalence and risk factors of postpartum depression (PPD) among tribal women who had recently delivered. A total of 100 postpartum tribal women who delivered at the RHTC during the study period were included. The participants were selected using a systematic random sampling technique from delivery records maintained at the RHTC to ensure a representative sample and reduce selection bias. Women who were referred to other healthcare centers due to pregnancy complications were excluded to maintain homogeneity of the study population. The sample size was calculated based on a previously reported PPD prevalence of 6% from Dubey *et al.* (2012) [16], using Cochrane's formula for sample size estimation. The calculation considered a 95% confidence interval and a 5% margin of error. The sample size came out to be 87. To account for potential non-response or incomplete data, a 10% contingency was added, resulting in a final sample size of 100 women. Data collection was conducted through home visits, a method chosen to enhance participation and accuracy by enabling face-to-face interaction in the participants' natural environment. Accredited Social Health Activists (ASHAs) accompanied the researchers to facilitate communication, especially to overcome language barriers and to build trust within the community. A pre-tested semi-structured questionnaire was used to collect detailed socio-demographic information such as age, education, family income and obstetric history including parity, mode of delivery and pregnancy complications. For screening PPD, the Edinburgh Postnatal Depression Scale (EPDS) [17], a widely validated 10-item tool, was administered. A cutoff score of 13 was used to identify mothers at risk for postpartum depression, consistent with established guidelines. Ethical approval was obtained from the Institutional Ethics Committee of the affiliated medical college. Written informed consent was obtained from all participants after explaining the study objectives and ensuring confidentiality of responses. Data were entered into Microsoft Excel and analyzed using the trial version of SPSS software. Descriptive statistics summarized the socio-demographic and clinical characteristics of the study population. Inferential statistical tests including chi-square tests and Fisher's exact tests were applied to examine associations between various factors and the presence of PPD. A p-value less than 0.05 were considered statistically significant, indicating a meaningful relationship between studied variables and postpartum depression. This study used a clear method to find out how common PPD is among tribal women in an underdeveloped area and what factors increase their risk. The goal was to understand their mental health needs and improve support for new mothers in similar settings.

## Results and Discussion:

Our study was conducted among 100 postpartum tribal women with a mean age of 23.5 ± 2.88 years, which is comparable to the mean age reported by Guin and Rawat in Jabalpur (24.5 years) [18] and Mathur *et al.* in Indore (24.5 years) [19]. Most of the participants in this study were illiterate (45%) or had only primary to middle school education (52%). About 44% of the women were engaged in agricultural labor and 26% were unemployed. A majority of families (58%) had a monthly income below INR 10,702. Using the Edinburgh Postnatal Depression Scale (EPDS) as a validated screening tool (cut-off ≥13), it was observed that 12% of participants were likely suffering from postpartum depression. As shown in Table 1 (see PDF) and Figure 1(see PDF), 55% of women had EPDS scores <9, 33% scored between 10-12 (suggestive of mild depressive symptoms) and 12% scored ≥13, indicating probable PPD. These findings are consistent with similar studies across India. Kapoor *et al.* in Eastern Uttar Pradesh reported a PPD proportion of 12.14% [20], Guin and Rawat observed 12.8% in Jabalpur, Madhya Pradesh [18] and Shriraam *et al.* documented 11% in rural Tamil Nadu [21]. However, Dubey *et al.* [16] and Mathur *et al.* [19] observed lower proportions of 6% in urban tertiary care centers in Delhi and Indore, respectively. Reproductive characteristics were found to be significantly associated with postpartum depression. In this study, women with higher gravida (>2 pregnancies) had significantly more depression (p = 0.022). Similarly, higher parity (p = 0.031), more than one live child (p = 0.019) and more than one total child (p = 0.012) were all significantly associated with increased PPD, as shown in Table 2 (see PDF). Unlike other studies, several socio-demographic and psychosocial variables were not significantly associated with depression in this study. No statistically significant association was observed for domestic violence, gender of the baby, marital status, or substance use by the husband. In contrast, Dubey *et al.* [16] identified factors such as family structure, marital conflict, lower socioeconomic status and birth of a female child as significant contributors to PPD. Shriraam *et al.* [21] found associations with age, education, adverse life events, domestic violence, alcohol use by the husband and poor relations with in-laws. Additionally, Guin and Rawat [18] highlighted unplanned pregnancies and lack of family support as important predictors.

## Conclusion:

12% of participants experienced postpartum depression, highlighting the significance of maternal and reproductive factors, such as gravida, parity and the number of children, in influencing PPD. Domestic Violence and Female child showed no clear impact on depression. Differences in significant factors between studies suggest that PPD is multifactorial and influenced by diverse social, cultural and demographic factors. These findings highlight the need for better support for mothers, especially regarding reproductive health and maternal wellbeing.
